# New Insights on the Genetic Basis Underlying SHILCA Syndrome: Characterization of the *NMNAT1* Pathological Alterations Due to Compound Heterozygous Mutations and Identification of a Novel Alternative Isoform

**DOI:** 10.3390/ijms22052262

**Published:** 2021-02-24

**Authors:** Víctor Abad-Morales, Ana Wert, María Ángeles Ruiz Gómez, Rafael Navarro, Esther Pomares

**Affiliations:** 1 Fundació de Recerca de l’Institut de Microcirurgia Ocular, 08035 Barcelona, Spain; wert@imo.es (A.W.); navarro@imo.es (R.N.); 2 Department of Genetics, Institut de Microcirurgia Ocular (IMO), 08035 Barcelona, Spain; 3 Department of Pediatric Ophthalmology, Strabismus and Neurophthalmology, Institut de Microcirurgia Ocular (IMO), 08035 Barcelona, Spain; 4 Pediatric Metabolic Unit and Neuropediatrics, Hospital Universitari Son Espases, 07120 Palma de Mallorca, Spain; ma.ruiz@ssib.es; 5 Department of Retina, Institut de Microcirurgia Ocular (IMO), 08035 Barcelona, Spain

**Keywords:** Leber congenital amaurosis (LCA), spondylo-epiphyseal dysplasia, sensorineural hearing loss, intellectual disability and Leber congenital amaurosis (SHILCA), nicotinamide mononucleotide adenylyltransferase 1 (*NMNAT1*), transcriptional alteration, inherited retinal diseases, macular coloboma, developmental delay, hypomyelination, sensorineural hearing loss, spondylo-epiphyseal dysplasia

## Abstract

This study aims to genetically characterize a two-year-old patient suffering from multiple systemic abnormalities, including skeletal, nervous and developmental involvements and Leber congenital amaurosis (LCA). Genetic screening by next-generation sequencing identified two heterozygous pathogenic variants in nicotinamide mononucleotide adenylyltransferase 1 (*NMNAT1*) as the molecular cause of the disease: c.439+5G>T and c.299+526_*968dup.This splice variant has never been reported to date, whereas pathogenic duplication has recently been associated with cases displaying an autosomal recessive disorder that includes a severe form of spondylo-epiphyseal dysplasia, sensorineural hearing loss, intellectual disability and LCA (SHILCA), as well as some brain anomalies. Our patient presented clinical manifestations which correlated strongly with this reported syndrome. To further study the possible transcriptional alterations resulting from these mutations, mRNA expression assays were performed in the patient and her father. The obtained results detected aberrant alternative transcripts and unbalanced levels of expression, consistent with severe systemic involvement. Moreover, these analyses also detected a novel *NMNAT1* isoform, which is variably expressed in healthy human tissues. Altogether, these findings represent new evidence of the correlation of *NMNAT1* and SHILCA syndrome, and provide additional insights into the healthy and pathogenic expression of this gene.

## 1. Introduction

Inherited retinal dystrophies (IRD) are a heterogeneous group of genetic rare diseases characterized by the progressive degeneration of the photoreceptor cells (i.e., rods and cones) and/or the retinal pigmented epithelium [1]. Leber congenital amaurosis (LCA) (ORPHA:65) is one of the earliest onset types of IRD, and is associated with severe visual impairment at birth or within the first years of life [2,3]. The incidence of LCA is 1:33,000–1:50,000 live births, and LCA accounts for 20% of blindness in school-age children and 5% of all IRD. Patients with the pathology experience dramatically reduced visual acuity or complete blindness, as well as nystagmus, photophobia and high hyperopia, among other symptoms. At a molecular level, LCA is highly genetically heterogeneous and, to date, mutations in at least 25 genes have been reported to cause this disease, typically as an autosomal recessive inherited trait, although some autosomal dominant cases have also been reported (RetNet, The Retinal Information Network) [4,5,6,7]. Even though these pathogenic variants are mostly related to a functional retinal impairment, LCA may also be linked to syndromes presenting with other features, such as neurodevelopmental delay, intellectual disability, oculomotor apraxia-type behavior or renal dysfunction [8,9]. This phenotypic complexity partly depends on the altered gene and the nature of the mutations detected in the patient; whether they are point mutations (missense, nonsense or splicing) or changes in the genetic dose (small or gross deletions, or insertions); and the concrete localization within the functional domains of the resulting protein. Many research studies have focused on the functional pathogenic impact of described mutations in the LCA genes [10,11].

A recent study described a novel LCA syndrome in three children from two unrelated Italian families, all three of whom displayed an autosomal recessive disorder that included a severe form of spondylo-epiphyseal dysplasia, sensorineural hearing loss, intellectual disability and Leber congenital amaurosis (SHILCA), as well as some brain anomalies [12]. Genetic analysis identified a shared homozygous *Alu*-mediated duplication in nicotinamide mononucleotide adenylyltransferase 1 (*NMNAT1*), which compromises the normal expression of this gene and leads to the production of aberrant mRNAs.

*NMNAT1* was first associated with LCA in 2012 [13,14,15], and, since then, more than 70 different pathogenic variants have been reported for this gene, mostly related to autosomal recessive LCA type 9 (LCA9) [16,17]. According to the UCSC Genome Browser database, three different transcript variants have been reported to date: variants 1 and 2 (NM_022787.3 and NM_001297778.1, respectively) encode isoform 1, whereas variant 3 (NM_001297779.1) encodes isoform 2. In this regard, although *NMNAT1* is variably expressed in all tissues and cell types (GTEx Portal), no comprehensive study of the expression levels for the different variants has been performed to date. At protein level, the NMNAT1 enzyme plays a key role in the biosynthesis of nicotinamide adenine dinucleotide (NAD), and is essential for the survival of eukaryotic cells [18,19]. However, despite the ubiquitous expression of NMNAT1, patients with LCA9 have no other systemic deficits outside the retina, as LCA9-associated mutant NMNAT1 proteins retain enzymatic activity and other biochemical functions, but appear to be less stable under conditions associated with cell stress [20].

In the present work, we report the first case of a patient presenting SHILCA syndrome due to compound heterozygosity of the *Alu*-mediated *NMNAT1* duplication and a novel splicing mutation. Furthermore, an extensive study of the transcriptional effects of these mutations on *NMNAT1* expression has also been performed, which identified alternative transcripts and altered levels of expression. Surprisingly, an unreported transcript was also detected in control individuals. This novel variant encodes a different isoform and is variably expressed in human tissues. Altogether, these findings represent new evidence of the correlation between the previously reported *NMNAT1* genomic rearrangement and SHILCA syndrome, and provide novel insights into the healthy and pathogenic expression of this gene.

## 2. Results

### 2.1. Clinical Presentation

**Figure 1:**
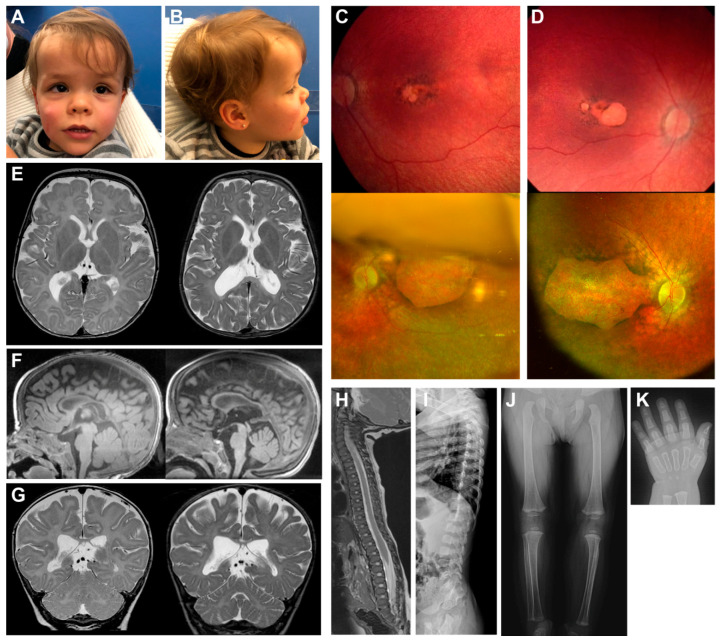
Clinical images of the patient. Frontal (**A**) and lateral (**B**) photographs of the patient’s head show facial appearances with mild coarsening and a deep nasal bridge. Retinographies of the left (**C**) and right (**D**) eyes present macular colobomatous involvement due to a retinal degeneration. Upper images were obtained at the age of six months whereas lower images were taken at age two years. Cerebral magnetic resonance imaging (MRI) (**E**–**G**) show hypomyelinating leukoencephalopathy, and progressive brain and cerebellum atrophy. Left images were obtained at the age of six months, whereas right images were obtained at age two years. Lateral spine MRI image and radiograph (**H**,**I**, respectively) present small epiphyseal changes due to a spondylo-epiphyseal dysplasia at six months of age. Skeletal radiographs (**J**,**K**) show severe ossification delay when the patient was 20 months old.

**Figure 2:**
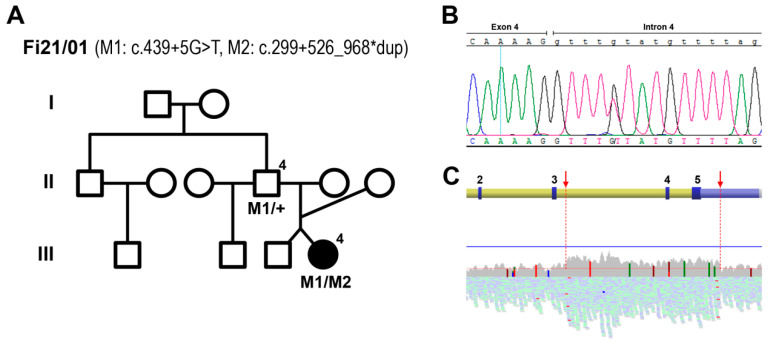
Genetic screenings allowed the discovery of the molecular cause of disease in the patient. Pedigree from family Fi21/01 (**A**) show the identified nicotinamide mononucleotide adenylyltransferase 1 (*NMNAT1*) pathogenic mutations M1: c.439+5G>T and M2: c.299+526_968*dup. The mutant allelic combination, analyzed by whole exome sequencing, is shown in the patient (individual III.4) and in the father (individual II.4, of Bulgarian origin). The confirmation of M1 was obtained by Sanger sequencing (**B**). The detection of the genetic rearrangement M2, corresponding to the duplication of exons 4 and 5 of *NMNAT1*, was detected by analyzing the BAM file from the whole genome sequencing of the patient (**C**). The upper scheme shows the genetic localization within the gene (exons 2 to 5, numbered), whereas the reading coverage is depicted in the lower graph. The red arrows show the region of the duplication, from the beginning of intron 3 to the middle of the 3′ UTR.

A two-year-old Spanish child (Figure 1) was born prematurely, together with an unaffected nonidentical twin, after 32 weeks and 5 days of gestation, from an ovum donation procedure (family Fi21/01: individual III.4, Figure 2A). In her fifth month of life, the child presented several systemic abnormalities, including developmental delay with cervicoaxial hypotonia, short stature (skeletal dysplasia), generalized dyskinesia and erratic eye movements with absent fixation.

Based on these clinical symptoms, the patient was studied by magnetic resonance imaging (MRI). The results showed severe hypomyelination and signs of brain atrophy, measured as brain volume loss, with mild ventricular expansion, subarachnoid space increase and severe corpus callosum hypoplasia (Figure 1). Follow-up examinations took place when the patient was two years old, at which time the patient’s height was 72 cm (*p* < 1, −4.72 SD), weight was 8.2 kg (*p* < 1, −2.97 SD) and head circumference was 49 cm (*p* = 82, 0.94 SD). These examinations revealed axial hypotonia with ataxia, a severe ossification delay, spondylo-epiphyseal dysplasia and mild sensorineural hearing loss. Moreover, facial appearance showed mild coarsening and a deep nasal bridge. The cranial MRI confirmed hypomyelinating leukoencephalopathy, although with few subcortical white matter myelinated islets, and progressive brain and cerebellum atrophy (Figure 1).

Regarding the visual exploration, the patient presented poor visual tracking with nystagmus and esotropia at the age of six months. No abnormalities were detected in the anterior chamber of both eyes, whereas the fundus examination showed mild optic disc pallor, marked attenuation of retinal vessels and widespread retinal pigmented epithelium alterations, with an absence of bone spicules. In the macular region, areas of geographic atrophy mimicking the colobomatous lesions were found (Figure 1). Moreover, an electroretinogram (ERG) showed scotopic and photopic response below noise levels. The exploration under cyclorefraction showed a high hypermetropia of +8.00 diopters (Dp) in both eyes. Altogether, the ocular clinical symptoms presented by the patient were consistent with those of LCA. After almost two years of follow-up, the patient’s best corrected visual acuity (BCVA) was light perception, and the retinographies showed an evolution of the retinal involvement, with increased areas of geographic macular atrophy in both eyes (Figure 1).

### 2.2. Genetic Analyses

The patient was analyzed using a whole-exome sequencing (WES) strategy focusing on a defined set of 30 LCA-related genes (Table S1). Only variants accomplishing frequency and deleterious criteria were considered as a possible pathogenic molecular cause (Table S2). From these results, the two heterozygous variants identified in *ALMS1* and *LCA5* were discarded due to inconclusive conservational and pathogenic predictions. Conversely, the heterozygous nucleotide variant c.439+5G>T in *NMNAT1* was predicted as pathogenic by the splicing bioinformatic tools, as it affects the exon 4 donor splice site of the gene, and thus, was considered for submission to ClinVar. This variant was further confirmed by Sanger sequencing (Figure 2B). However, considering autosomal recessive as the most suitable inheritance pattern, based on the *NMNAT1* pathological precedents and in consonance with the family tree, this mutation was insufficient to be the molecular cause of the pathology. The BAM file resulting from the WES analysis of the patient was analyzed. An increase in reads involving two of the five exons of *NMNAT1* (exons 4 and 5, NM_022787.3) could be detected, consistent with a heterozygous duplication of this region. For this reason, whole genome sequencing (WGS) was performed to further determine the concrete spanning of the putative duplication. The results showed a higher mean coverage along 7.4 kb within *NMNAT1*, from the beginning of intron 3 to the middle of the 3′ UTR (Figure 2C). The rearrangement boundaries were then amplified and Sanger sequencing was performed using specific primers to confirm the presence of the heterozygous duplication, which coincided with the mutation recently reported as the cause of the novel SHILCA syndrome (NM_022787.3:c.299+526_*968dup) [12].

The study of the father’s DNA (individual II.4, Figure 2A) by PCR amplification determined that he only presented the heterozygous variant c.439+5G>T. Thus, the splicing mutation and the duplication detected in the patient were present in heteroallelic combination, in accordance with the proposed inheritance pattern and the familial history (Figure 2A).

### 2.3. NMNAT1 Expression

**Figure 3:**
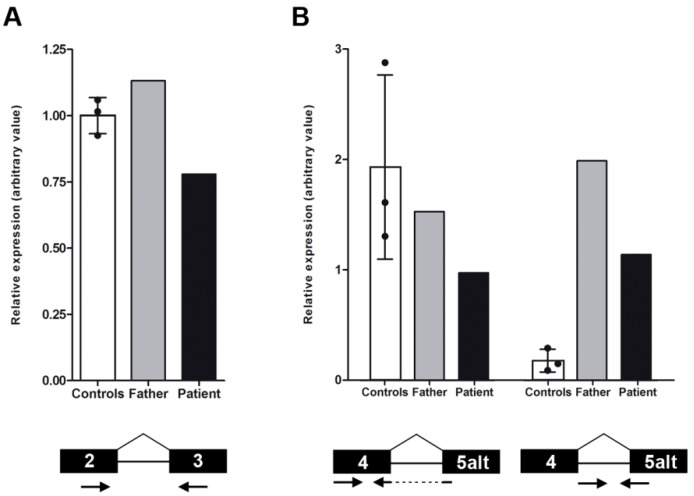
Study of the nicotinamide mononucleotide adenylyltransferase 1 (*NMNAT1*) expression levels by real-time reverse transcriptase (RT)-PCR was performed in the patient and her father, and compared with those of three healthy control subjects. The first graph (**A**) shows slightly higher levels of total *NMNAT1* mRNA in the father and lower levels in the patient. The second graph (**B**) represents the level of two concrete *NMNAT1* transcript variants: the alternative isoform (transcript variant NM_001297779.1, left bars) and a newly identified isoform with the intron 4 retention (right bars). Only the patient showed decreased expression of the alternative isoform, whereas both tested family members presented dramatically higher levels of expression of the new isoform. In all cases, control error bars correspond to the standard deviation between the three biological replicates. The concrete amplicon localization is shown under the graph in each condition. Arrows represent forward and reverse primers, whereas the black boxes are the concrete target exons.

In order to detect the possible *NMNAT1* transcriptional alterations produced by the discovered mutations, mRNA expression assays were performed on cDNA samples from both the patient and her father. PCR amplification of the two known *NMNAT1* isoforms (isoform 1 or canonical, and 2 or alternative) detected in the patient an aberrant transcript carrying the duplication of exon 4 and partially exon 5, as already reported [12]. Moreover, two novel transcript variants were also detected: the alternative isoform with the retention of intron 4, present in both individuals; and the canonical isoform skipping exon 4, only faintly detected in the father. Following these findings, real-time reverse transcriptase (RT)-PCR assays were performed to further quantify the levels of expression of the identified *NMNAT1* transcript variants, comparing each individual’s samples with three unrelated healthy subjects used as controls (Figure 3). The amplification of exons 2 and 3, which are present in all the *NMNAT1* transcript variants, showed a decreased expression in the patient, whereas the father presented an increased signal compared with the controls (Figure 3A). Following these findings, the authors planned to assess the levels of expression of all the different detected isoforms in the patient. Unfortunately, as a consequence of the aberrant transcript carrying exons 4 and 5 duplication, no real-time PCR could be performed to measure the expression of the canonical isoform. However, regular PCR amplification with specific primers designed to solely detect the canonical isoform (Table S3), excluding the aberrant duplication, confirmed some expression of this isoform in the patient (Figure S1).

Regarding the other two isoforms (alternative and the intron 4 retention), specific real-time PCRs were performed in the patient and her father, together with the control subjects. Only the patient presented decreased expression of the alternative isoform, whereas the father’s value was lower than that of the controls’, but was within normal limits (Figure 3B). In contrast, the presence of mRNA with the retention of intron 4 was highly increased in the patient and her father, in consonance with an alteration of the *NMNAT1* transcriptional splicing. Surprisingly, the expression of this isoform was also detected in two of the three control subjects, although at significantly lower levels. The authors hypothesized that this novel isoform was also normally expressed in healthy cells.

**Figure 4:**
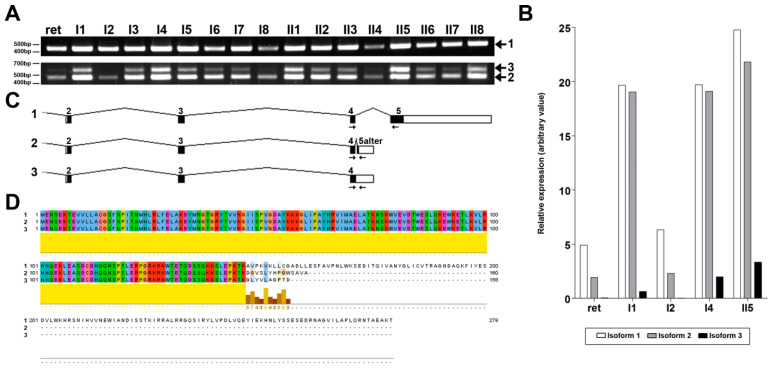
Transcriptional study of the different nicotinamide mononucleotide adenylyltransferase 1 (*NMNAT1*) isoforms in human tissues. PCR amplification with specific primers (Table S3) using a panel of human tissues (**A**) detected variable ubiquitous expressions of three different isoforms: canonical or 1 (transcript variants NM_022787.3 and NM_001297778.1, upper panel), alternative or 2 (transcript variant NM_001297779.1, lower panel, bottom band) and a newly described isoform 3 (lower panel, top band). ret: retina. I1: liver. I2: skeletal muscle. I3: kidney. I4: pancreas. I5: heart. I6: brain. I7: placenta. I8: lung. II1: ovary. II2: small intestine. II3: colon. II4: leukocyte. II5: spleen. II6: thymus. II7: prostate. II8: testis. In order to quantify these expressions, real-time reverse transcriptase (RT)-PCR assays were performed for some of these human tissues (**B**). Levels of expression of isoform 1 (white bars) were higher in all tested tissues, followed by isoform 2 (gray bars). The novel isoform 3 presented lower levels of expression, although it was always detected. (**C**)The sequence of coding exons for each isoform were created using GeneDrawer software (Insilicase). Primer pairs used for PCR amplification (seen in **A**) are represented with black arrows in each isoform. (**D**)The alignment of the resulting protein sequences (in order: isoforms 1, 2 and 3, as indicated within the figure), using Jalview software [21], showed an identical sequence along the first 146 amino acids and some conservation of the last residues (lower yellow line).

To this end, a comprehensive study was performed using a panel of cDNAs from a variety of human tissues, amplifying the canonical and the alternative *NMNAT1* isoforms. The obtained results showed a ubiquitous variable expression of the two reported isoforms in all tissues, together with another amplicon present in the alternative isoform amplification (Figure 4A). Sanger sequencing of this band confirmed the expression of the newly detected alternative isoform with intron 4 retention, as previously identified by real-time PCR (Figure 4C). Furthermore, real-time PCR analyses were performed in some of the human tissues, to relatively quantify each isoform. Although the expression levels were variable, the canonical isoform was always more highly detected, whereas isoform 3 presented the lowest levels in all tested tissues (Figure 4B). The alignment of the predicted proteins resulting from the three isoforms revealed a common sequence of 146 amino acids, corresponding to exons 2, 3 and 4, and relatively conserved C-terminal of the last residues of the two shorter isoforms (Figure 4D).

## 3. Discussion

In this study, we present a two-year-old patient suffering from multiple systemic abnormalities, including developmental delay, axial hypotonia with ataxia, severe ossification delay, spondylo-epiphyseal dysplasia, distinctive facial appearances, sensorineural hearing loss, hypomyelinating leukoencephalopathy, progressive brain and cerebellum atrophy, and LCA. Based on the ocular phenotype, an extensive genetic screening was performed in the patient, allowing the identification of two heterozygous pathogenic variants in *NMNAT1* as the molecular cause of the LCA: c.439+5G>T and c.299+526_*968dup. The first pathogenic variant, which had never been previously reported, was also detected in heterozygosity in the father, who is of Bulgarian origin. On the other hand, the heterozygous identified duplication has recently been described as the genetic cause of the novel LCA syndrome named SHILCA [12]. As reported, patients with this syndrome displayed an autosomal recessive disorder that included a severe form of spondylo-epiphyseal dysplasia, sensorineural hearing loss, intellectual disability and LCA, as well as some brain anomalies. The clinical manifestations of our patient correlated strongly with those of reported SHILCA cases, including ocular, nervous and skeletal affectations. The retinal involvement was almost identical, with mild optic disc pallor, marked attenuation of retinal vessels and characteristic macular colobomatous atrophy, as already described in other LCA patients [22]. However, the severity of the pathology appeared to be milder for some traits, as was the case regarding the mild sensorineural hearing loss. Moreover, concrete symptoms, such as the intellectual disability or the biconcave spine profile and scoliosis, were not detected in our case. In this regard, although the early age of the patient does not allow a proper evaluation of the evolution and severity of the pathology, these differences could be attributed to the presence of the c.439+5G>T variant in one allele, instead of homozygous duplication. In any case, our findings are crucial to correlate the *NMNAT1* genetic alterations identified in our patient with the other detected phenotypic anomalies, as well as to confirm the autosomal recessive pattern of inheritance.

In the work by Bedoni et al. [12], haplotype analyses revealed rare heterozygous single nucleotide polymorphism (SNP) genotypes in the proximity of the duplication, which were found in the three Italian patients. This identical haplotype surrounding the rearrangement was also detected in an additional Spanish patient, indicative of a common and likely remote ancestral genetic event. In our case, the duplication was supposedly inherited via the maternal allele. Although the patient was conceived by ovum donation, it is possible that the donor could be from the Mediterranean region, which would confirm the event of a founder mutation.

At the transcriptional level, the study of possible implications of these mutations in *NMNAT1* expression allowed us to identify significant alterations, including aberrant transcription events and impaired mRNA levels. The total *NMNAT1* expression in the patient was decreased by approximately 25% compared with that of the control subjects. Likewise, the mRNA levels of isoform 2 were also reduced in the patient. Considering that these events were not detected in the father, we propose that these transcriptional alterations were a consequence of the *NMNAT1* duplication, in accordance with the reported reduction of wild-type expression by at least three times compared to that in the homozygous SHILCA individuals [12]. In contrast, isoform 3 was substantially increased in both the patient and her father, versus the level seen in the control subjects. Thus, it could be assumed that the increment of the intron 4 retention isoform is a direct molecular effect of the c.439+5G>T mutation. In fact, higher levels of the total amount of *NMNAT1* mRNA were also observed in the father, which could probably be attributed to this enhanced isoform 3 expression. Altogether, the c.299+526_*968dup mutation decreases the levels of both isoforms 1 and 2, whereas the c.439+5G>T mutation alters the relative expression of *NMNAT1* isoforms. These alterations are most probably responsible for the multisystem involvement instead of a retina-restricted degeneration, as they compromise the correct *NMNAT1* expression by unbalancing the relative levels of all isoforms and reducing the expression of the canonical variant.

In addition to the transcriptional alteration attributed to the pathogenic *NMNAT1* mutations identified in the patient, a new isoform was also described in healthy controls. This novel coding mRNA is also ubiquitously expressed with variable levels, albeit at levels significantly lower than that of the two known isoforms, and presents a protein sequence very similar to that of isoform 2. These results could be of major importance in the molecular mechanisms of the NMNAT1 enzyme, and could even involve other novel functions yet to be discovered. Further experimental approaches are needed to characterize the functional role of this novel *NMNAT1* isoform.

In conclusion, to our knowledge, our patient is the first case with SHILCA syndrome caused by the compound heterozygosity of the already reported *Alu*-mediated *NMNAT1* duplication and a novel splicing pathogenic variant. Herein, we provide new evidence of the correlation between this duplication and SHILCA. The transcriptional study of the possible alterations identified alternative transcripts and unbalanced levels of expression, correlating with the hypothesis that severe mutations in this gene are associated with multisystem—instead of retinal-restricted—phenotypes. Moreover, an unreported alternative transcript was also ubiquitously detected with variable levels of expression in different healthy human tissues. All these results contributed to determine the pathogenic impact of the identified causative mutations, to provide novel insights into the healthy and pathogenic expression of this gene and to increase the knowledge of the molecular basis underlying the SHILCA syndrome, to further improve the diagnosis, precise prognosis, genetic counseling and future proper treatments for this disease.

## 4. Materials and Methods

### 4.1. Clinical Examinations

The patient in this study was clinically diagnosed at the Institut de Microcirurgia Ocular (IMO) (Barcelona, Spain) and the Hospital Universitari Son Espases (Palma de Mallorca, Spain). Standard ophthalmic examinations were performed, such as best corrected visual acuity (BCVA), fundus retinography, full-field electroretinography (ffERG) and perimetry, among others. Moreover, other systemic evaluations were also carried out, including MRI and radiographic images. The study was conducted according to the guidelines of the Declaration of Helsinki, and approved by the Ethics Committee of IMO (protocol code 160321_96, date of approval 29 March 2016).

### 4.2. Genetic Screenings

A peripheral blood sample was obtained from the patient and her father, and placed in EDTA tubes. Automated extraction of genomic DNA was performed using the KingFisher Duo Prime purification system (Thermo Fisher Scientific, Waltham, MA, USA). The patient’s DNA was analyzed by whole exome sequencing (WES), focusing on a panel of 30 genes related to LCA (Table S1), and the results were filtered according to deleterious potential and minor allele frequency, as previously reported [23]. Putative pathogenic variants were confirmed by Sanger sequencing. Moreover, whole genome sequencing (WGS) of the patient’s DNA was also performed using the Illumina platform and the TruSeq DNA PCR-Free library kit (Illumina, San Diego, CA, USA), obtaining an average depth of 39× and a fragment length median of 390 bp. The study of gross genomic rearrangements within the LCA genes was carried out by analyzing sequencing reads in BAM files from WES and WGS with the Alamut Visual software v2.11 (SOPHiAGENETICS, Boston, MA, USA). Moreover, PCR confirmation of the *NMNAT1* exon 4 and 5 duplication was performed using a forward primer located in the 3′UTR and a reverse primer matching the intron 3 (Table S3). Finally, the familial origin of the detected pathogenic variants was established based on segregation analyses using the DNA sample from the father.

### 4.3. Expression Assays

Total RNA from the patient and her father was obtained from 500 μL whole blood in 1.3 mL RNA*later* by using the RiboPure-Blood purification kit (Thermo Fisher Scientific, Waltham, MA, USA), according to the manufacturer’s instructions. The cDNA chains were obtained by reverse transcription using the Transcriptor High Fidelity cDNA Synthesis Kit (Roche Diagnostics, Basilea, Switzerland). Exon-exon *GAPDH* amplification was used as a control for the detection of genomic DNA contamination. The cDNA samples were then used to assess the differential expression of *NMNAT1* transcript variants by PCR amplification of the coding regions of both isoforms (isoform 1 or canonical: NM_022787.3 and NM_001297778.1; and isoform 2 or alternative: NM_001297779.1) using the primers listed in Table S3. Moreover, real-time RT-PCR assays were performed to quantify the expression levels of the different transcripts detected in the patient and her father, and both samples were compared with those of three unrelated healthy controls. For the analysis of total *NMNAT1* expression, a common region for all transcript variants (exon 2–3 boundary, Hs00978910_g1) was selected for amplification using TaqMan Gene Expression Assay (Applied Biosystems, Foster City, CA, USA). Relative gene expression was assayed in four replicates, using the housekeeping gene *GAPDH* expression for normalization (Hs99999905_m1), after confirming its stable expression throughout the different samples in comparison to two other reference genes (*ACTB* and *B2M*). Specific primer pairs were used to target each *NMNAT1* variant (Table S3) using the PowerUp SYBR Green Master Mix assay (Applied Biosystems, Foster City, CA, USA) and *GAPDH* expression for normalization. All real-time RT-PCR reactions were performed on a QuantStudio 3 instrument (Applied Biosystems, Foster City, CA, USA).

Moreover, the expression levels of the canonical, alternative and novel *NMNAT1* isoforms (1, 2 and 3, respectively) were studied using the commercial retinal PCR Ready First Strand cDNA (BioChain, San Francisco Bay Area, CA, USA) and MTC cDNA Panels I and II (Clontech Laboratories, Mountain View, CA, USA) as templates with specific primer pairs (Table S3). Although MTC Panels were commercially normalized, the saturating PCR conditions (35 cycles) prevented the proper quantification of the amplified bands. For this reason, a real-time RT-PCR was performed with some cDNA tissue samples using the conditions mentioned above.

## Data Availability

The data presented in this study are available on request from the corresponding author. The data are not publicly available due to privacy restrictions.

## Supplementary Materials

The following are available online at https://www.mdpi.com/1422-0067/22/5/2262/s1, Table S1: List of the 30 genes included in the LCA panel. Table S2: Putative pathogenic variants detected by WES in the LCA genes. Table S3: List of primers used in the NMNAT1 expression assays. Figure S1: Study of the *NMNAT1* canonical isoform expression.

## References

[1] Rattner A., Sun H., Nathans J. (1999). Molecular Genetics of Human Retinal Disease. Annu. Rev. Genet..

[2] Tsang S.H., Sharma T. (2018). Leber Congenital Amaurosis. Advances in Experimental Medicine and Biology.

[3] Allikmets R. (2004). Leber congenital amaurosis: A genetic paradigm. Ophthalmic Genet..

[4] Kondkar A.A., Abu-Amero K.K. (2019). Leber congenital amaurosis: Current genetic basis, scope for genetic testing and personalized medicine. Exp. Eye Res..

[5] Koenekoop R.K., Lopez I., Allikmets R., Cremers F.P., Hollander A.I.D. (2008). Genetics, phenotypes, mechanisms and treatments for Leber congenital amaurosis: A paradigm shift. Expert Rev. Ophthalmol..

[6] Hollander A.I.D., Roepman R., Koenekoop R.K., Cremers F.P. (2008). Leber congenital amaurosis: Genes, proteins and disease mechanisms. Prog. Retin. Eye Res..

[7] Cremers F.P.M., Hurk J.A.J.M.V.D., Hollander A.I.D. (2002). Molecular genetics of Leber congenital amaurosis. Hum. Mol. Genet..

[8] Ronquillo C., Bernstein P.S., Baehr W. (2012). Senior–Løken syndrome: A syndromic form of retinal dystrophy associated with nephronophthisis. Vis. Res..

[9] Peter V.G., Quinodoz M., Pinto-Basto J., Sousa S.B., Di Gioia S.A., Soares G., Leal G.F., Silva E.D., Msc R.P.G., Miyake N. (2019). The Liberfarb syndrome, a multisystem disorder affecting eye, ear, bone, and brain development, is caused by a founder pathogenic variant in the PISD gene. Genet. Med..

[10] Sheck L., Davies W.I.L., Moradi P., Robson A.G., Kumaran N., Liasis A.C., Webster A.R., Moore A.T., Michaelides M. (2018). Leber Congenital Amaurosis Associated with Mutations in CEP290, Clinical Phenotype, and Natural History in Preparation for Trials of Novel Therapies. Ophthalmology.

[11] Astuti G.D.N., Bertelsen M., Preising M.N., Ajmal M., Lorenz B., Faradz S.M.H., Qamar R., Collin R.W.J., Rosenberg T., Cremers F.P.M. (2016). Comprehensive genotyping reveals RPE65 as the most frequently mutated gene in Leber congenital amaurosis in Denmark. Eur. J. Hum. Genet..

[12] Bedoni N., Quinodoz M., Pinelli M., Cappuccio G., Torella A., Nigro V., Testa F., Simonelli F., Corton M., Lualdi S. (2020). An Alu-mediated duplication in NMNAT1, involved in NAD biosynthesis, causes a novel syndrome, SHILCA, affecting multiple tissues and organs. Hum. Mol. Genet..

[13] Falk M.J., Zhang Q., Nakamaru-Ogiso E., Kannabiran C., Fonseca-Kelly Z., Chakarova C., Audo I., Mackay D.S., Zeitz C., Borman A.D. (2012). NMNAT1 mutations cause Leber congenital amaurosis. Nat. Genet..

[14] Koenekoop R.K., Wang H., Majewski J., Wang X., Lopez I., Ren H., Chen Y., Li Y., Fishman G.A., Genead M. (2012). Mutations in NMNAT1 cause Leber congenital amaurosis and identify a new disease pathway for retinal degeneration. Nat. Genet..

[15] Chiang P.-W., Wang J., Chen Y., Fu Q., Zhong J., Chen Y., Yi X., Wu R., Gan H., Shi Y. (2012). Exome sequencing identifies NMNAT1 mutations as a cause of Leber congenital amaurosis. Nat. Genet..

[16] Coppieters F., Todeschini A.L., Fujimaki T., Baert A., De Bruyne M., Van Cauwenbergh C., Verdin H., Bauwens M., Ongenaert M., Kondo M. (2015). Hidden Genetic Variation in LCA9-Associated Congenital Blindness Explained by 5′UTR Mutations and Copy-Number Variations of NMNAT1. Hum. Mutat..

[17] Khan A.O., Budde B.S., Nürnberg P., Kawalia A., Lenzner S., Bolz H.J. (2018). Genome-wide linkage and sequence analysis challenge CCDC66 as a human retinal dystrophy candidate gene and support a distinct NMNAT1 -related fundus phenotype. Clin. Genet..

[18] Magni G., Amici A., Emanuelli M., Raffaelli N., Ruggieri S. (1999). Enzymology of NAD+ synthesis. Adv. Enzymol. Relat. Areas Mol. Biol..

[19] Berger F., Ramírez-Hernández M.H., Ziegler M. (2004). The new life of a centenarian: Signalling functions of NAD(P). Trends Biochem. Sci..

[20] Sasaki Y., Margolin Z., Borgo B., Havranek J.J., Milbrandt J. (2015). Characterization of Leber Congenital Amaurosis-associated NMNAT1 Mutants. J. Biol. Chem..

[21] Waterhouse A.M., Procter J.B., Martin D.M.A., Clamp M., Barton G.J. (2009). Jalview Version 2--a multiple sequence alignment editor and analysis workbench. Bioinformatics.

[22] Özgül R.K., Bozkurt B., Kıratlı H., Oğüş A., Kiratli H. (2005). Exclusion of LCA5 locus in a consanguineous Turkish family with macular coloboma-type LCA. Eye.

[23] Abad-Morales V., Burés-Jelstrup A., Navarro R., Ruiz-Nogales S., Méndez-Vendrell P., Corcóstegui B., Pomares E. (2019). Characterization of the cone-rod dystrophy retinal phenotype caused by novel homozygous DRAM2 mutations. Exp. Eye Res..

