# Supplementary material for: New Insights on the Genetic Basis Underlying SHILCA Syndrome: Characterization of the NMNAT1 Pathological Alterations Due to Compound Heterozygous Mutations and Identification of a Novel Alternative Isoform
Source: Int J Mol Sci. 2021 Feb 24;22(5):2262. doi: 10.3390/ijms22052262 (PMC7956282; doi:10.3390/ijms22052262)
Supplement: Supplementary file 1 [file ijms-22-02262-s001.zip › Table S2.docx]

| **Table S2.** Putative pathogenic variants detected by WES in the LCA genes. | | | | | | | | |
| --- | --- | --- | --- | --- | --- | --- | --- | --- |
| **Gene** | **GenBank ID** | **Position (GRCh38/hg38)** | **Nucleotide change** | **Amino acid change** | **Zygosity** | **Variant Effect** | **Existing Variation** | **MAF** |
| *ALMS1* | NM_015120.4 | chr2:73422928 | c.718C>T | p.Pro240Ser | Hetero | missense variant | rs1320912703 | 0,000008 (GnomAD_exome) |
| *LCA5* | NM_181714.3 | chr6:79487263 | c.1835G>C | p.Ser612Thr | Hetero | missense variant | rs764235088 |  |
| *NMNAT1* | NM_022787.3 | chr1:9981175 | c.439+5G>T | p.? | Hetero | splice region variant |  |  |
| Hetero: heterozygous; MAF: minor allele frequency. | | | | | | | | |
