# Supplementary material for: New Insights on the Genetic Basis Underlying SHILCA Syndrome: Characterization of the NMNAT1 Pathological Alterations Due to Compound Heterozygous Mutations and Identification of a Novel Alternative Isoform
Source: Int J Mol Sci. 2021 Feb 24;22(5):2262. doi: 10.3390/ijms22052262 (PMC7956282; doi:10.3390/ijms22052262)
Supplement: Supplementary file 1 [file ijms-22-02262-s001.zip › Table S3.docx]

| **Table S3.** List of primers used in the *NMNAT1* expression assays. | | |
| --- | --- | --- |
| **Name** | **Sequence** | **Target isoform** |
| ex2F | TCCGAGAAGACTGAAGTGGTTC | 1, 2, 3 |
| ex5R | CACAGCAGCTTGACCTTTGGC | 1 |
| ex5alterR | AGATCATGCCACTGCACTCCA | 2, 3 |
| ex5R2 | TTCTTGGACAAGATCTGGTACCA | 1 |
| int4F | TTAGCAGGACCCACGGACTA | 3 |
| int4R | GGTGATAGAGCGAGACTCCATCTA | 3 |
| ex5UTR F | GAGGCAGAGGTTGCAGTAAGC | duplication |
| int3R | CCATGATCAAGCAGCACTGGAT | duplication |
| NMNAT1 isoforms [1: NM_022787.3 and NM_001297778.1; 2: NM_001297779.1; 3: novel; duplication: c.299+526_*968dup] | | |
