# Supplementary material for: New Insights on the Genetic Basis Underlying SHILCA Syndrome: Characterization of the NMNAT1 Pathological Alterations Due to Compound Heterozygous Mutations and Identification of a Novel Alternative Isoform
Source: Int J Mol Sci. 2021 Feb 24;22(5):2262. doi: 10.3390/ijms22052262 (PMC7956282; doi:10.3390/ijms22052262)
Supplement: Supplementary file 1 [file ijms-22-02262-s001.zip › Figure S1.docx]

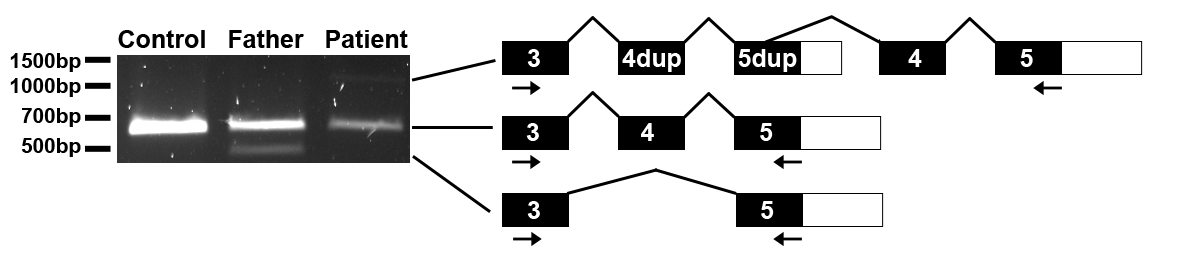


**Figure S1.** Study of the expression of the *NMNAT1* cannonical isoform (isoform 1) in the father and the patient, in comparison to a control subject, using primers specifically designed to detect the expression of this isoform (Table S3). The expression of isoform 1 was detected both in the father and the patient, although the later with significant lower levels. Moreover, two other alternative transcripts were also detected: the skipping of exon 4 in the father, and the duplication of exon 4 and partly exon 5 in the patient. The right panel represents the exon composition of the three detected mRNAs, together with the location of the primer pair (arrows).
